# Evaluation of the quality of COVID-19 prevention and control by a novel comprehensive evaluation model in a tertiary general hospital: a prospective observational study

**DOI:** 10.1186/s12889-021-12032-9

**Published:** 2021-11-06

**Authors:** Xiao Zhong, Dong-Li Wang, Lan-Fang Mo, Wen Zhang, Li-Hua Xiao, Xiang-Lin Wu, Yan-Wei Chen, Lei Yang

**Affiliations:** 1 Tertiary-grade & A-class office, Shenzhen Hospital, University of Chinese Academy of Sciences, Shenzhen, 518106 Guangdong China; 2 Testing center, Guangming District Center for Disease Control and Prevention, Shenzhen, Guangdong China

**Keywords:** Quality of COVID-19 prevention and control, Multi-index comprehensive evaluation, Subjective and objective combined weight, Efficacy coefficient method, TOPSIS method, Rank-sum ratio method, Grey relationship analysis method

## Abstract

**Background:**

Prevention and control (P&C) of Corona Virus Disease 2019 (COVID-19) is still a critical task in most countries and regions. However, there are many single evaluation indexes to assess the quality of COVID-19 P&C. It is necessary to synthesize the single evaluation indexes reasonably to obtain the overall evaluation results.

**Methods:**

This study was divided into three steps. Step 1: In February 2020, the improved Delphi method was used to establish the quality evaluation indexes system for COVID-19 P&C. Step 2: in March 2020, the CRITIC method was used to adjust the Order Relation Analysis (G1) method to obtain the subjective and objective (S&O) combination weights. The comprehensive evaluation value was obtained using the weighted Efficacy Coefficient (EC) method, weighted TOPSIS method, weighted rank-sum ratio (RSR) method, and weighted Grey Relationship Analysis (GRA) method. Finally, the linear normalization method was used to synthesize the evaluation values of different evaluation methods. Step 3: From April 2020 to May 2021, this evaluation method was used to monitor and assess COVID-19 P&C quality in critical departments prospectively. The results were reported to the departments monthly.

**Result:**

A quality evaluation indexes system for COVID-19 P&C was established. Kendall’s consistency test shows that the four evaluation method had good consistency (χ^2^ = 43.429, *P*<0.001, Kendall’s consistency coefficient = 0.835). The Spearman correlation test showed that the correlation between the combined evaluation results and the original method was statistically significant(*P* < 0.001). According to the Mann-Kendall test, from March 2020 to May 2021, the mean value of COVID-19 P&C quality in all critical departments showed an upward trend (*P* < 0.01).

**Conclusions:**

The combined comprehensive evaluation method based on the S&O combined weight was more scientific and comprehensive than the single weighting and evaluation methods. In addition, monitoring and feedback of COVID-19 P&C quality were helpful for the improvement of P&C quality.

## Background

The COVID-19 pandemic has been the world’s largest infectious disease pandemic in decades [1, 2]. At present, the COVID-19 epidemic in China has been controlled and entered the regular prevention and control (P&C) stage of “Outside defense import, inside defense rebound” [3]. However, the COVID-19 epidemic in most countries and regions outside China is still severe. By April 25, 2021, more than 146 million confirmed cases of COVID-19 [4] had been reported outside China, more than 1500 times the total number of confirmed cases in China. The success of China’s fight against COVID-19 is related to the implementation of scientific P&C measures, ranging from a province or a city to a community or a medical institution.

From the perspective of a medical institution, to do well in COVID-19 P&C, it should first understand the current situation of COVID-19 P&C in their institutions. Then, it is easy to grasp the key and take targeted improvement measures to continuously improve work quality, which requires regular evaluation of the quality of P&C work. However, there are many indicators to evaluate COVID-19 P&C work. For example, there are process indicators, including hand hygiene (HH) compliance rate, disinfection qualification rate, personal protective equipment (PPE) reserve complete rate, complete rate of protective facilities, COVID-19 P&C knowledge training rate, COVID-19 P&C knowledge awareness rate, the compliance rate of various COVID-19 P&C measures implementation. There are also outcome indicators, including the incidence of nosocomial infection, occupational exposure, and adverse events. The values of multiple indicators vary from high to low, and it isn’t easy to evaluate the overall quality of work only by comparing a single index. Therefore, a comprehensive evaluation of all indicators is needed to comprehensively and accurately reflect COVID-19 P&C work quality.

The comprehensive evaluation method refers to a systematic and standardized method to evaluate multiple indexes and units simultaneously. It is generally divided into three steps: 1. Select evaluation indexes and establish an evaluation index system. Whether the comprehensive evaluation result is objective and accurate depends on whether the comprehensive evaluation index is accurate and comprehensive. Therefore, the selection of an evaluation index is an essential basic work in a comprehensive evaluation. 2. The evaluation indicators should be changed to dimensionless and trendless to eliminate the influence of dimensions and trends. 3. Determine the weights of evaluation indicators. The index is weighted by weight to reflect the relative importance of the index. 4. Summary and synthesis of comprehensive indicators. According to the standard value, evaluation method, and each indicator’s weight in the index system, each evaluation object is summarized and synthesized into an index, and the value of the synthesis index is compared to judge the quality of each object.

The application field and scope of comprehensive evaluation are vast. It is widely used in natural science, social science, and economics to evaluate the characteristics and properties of various things. Such as comprehensive evaluation of environmental monitoring, drug clinical trials, social security, quality of life, economic benefits, and production mode.

It also plays a vital role in health management. During the COVID-19 pandemic, some literature reported the issue of prioritizing COVID-19 patients using multi-indicator decision-making. For example, Mohammed Ma et al. [5] used Entropy-TOPSIS method for evaluation and benchmarking methodology to select the best classifiers for COVID-19 diagnosis. Majumder P et al. [6] presented a real-time death assessment monitoring of COVID-19 using TOPSIS MCDM to choose the most critical risk factors and applied GMDH to estimate death value within all confirmed cases. Singh R, et al. [7] suggested that many activities should not be performed during the COVID 19 pandemic period. These activities were considered as criteria and then applied to an analytic hierarchy process (AHP) to calculate their weights and assigned ranks/priorities according to their effect. Albahri As et al. [8] used AHP and Vikor to get COVID-19 patient prioritization dependent on their health conditions.

Furthermore, some methods had been reported in the literature to evaluate nosocomial infection P&C quality comprehensively. For example, Yun Z [9] evaluated hospital infection P&C quality using the relative distance and AHP. Suner A et al. [10] collected expert opinions regarding the criteria influencing the best HH preference. Afterward, these opinions were examined with the Multi-Attribute Utility Theory (MAUT) and the AHP. The result indicated that rubbing the hands with ABAS was the most favorable choice for IDCM specialists to prevent nosocomial infection. Xiao-meng C [11] evaluated ICU target monitoring in a hospital from 2010 to 2016 by the RSR method. However, these studies listed above used only a single weighting method and one or two results synthesis methods.

In the comprehensive evaluation process, the relative importance of indicators, i.e., the index’s weight, significantly influences the results. The determination methods of the index’s weight can be divided into subjective and objective weighting methods [12]. The subjective weighting method takes complete account of the personal wills of the evaluators but ignores the nature of the data. Therefore, in some cases, the evaluation objects can’t be effectively distinguished. The objective weighting method fully considers the nature of data, such as correlation and difference. However, it lacks subjective control and often produces results inconsistent with the facts. Therefore, only a good combination of S&O weights can reflect the evaluation’s comprehensiveness and scientificity. In addition, different results may be obtained due to different principles of various evaluation methods and different sensitivities of indicators to evaluation methods. Therefore, it is inappropriate to use only one evaluation method. At present, it is the development trend to apply various methods in a comprehensive evaluation [13].

Therefore, in this study, the combined evaluation methods weighted by S&O combined weights were put forward to improve the scientific nature of evaluation and the stability of results. Using this method to evaluate the quality of COVID-19 P&C, the overall situation and focus of COVID-19 P&C work can be grasped more scientifically and accurately, and targeted measures can be taken to improve the quality of COVID-19 P&C work continuously. This study will share an example of a tertiary general hospital in Shenzhen, China, using this approach to assess and improve the quality of COVID-19 P&C work since the COVID-19 pandemic. Furthermore, it provides a reference for medical institutions to do well in COVID-19 P&C.

## Methods

### Study institutions and design

The study was carried out from February 2020 to May 2021 in a tertiary general hospital with 1350 beds, 1115 doctors, and 1334 nurses in Guangming District, Shenzhen, Guangdong, China. It was divided into three steps. The first step was the establishment step of the evaluation indexes system: In February 2020, all single indicators for evaluating the quality of COVID-19 P&C were first listed, questionnaires were made, representative evaluation indicators were screened by the Delphi method [14], and an evaluation indexes system for the quality of COVID-19 P&C was established. The second step was the establishment of the evaluation method step. In March 2020, the evaluation indexes data were collected, and the objective weight obtained from the CRITIC method was used to adjust the G1 subjective weighting method. As a result, the S&O combined weights were obtained.

**Fig. 1:**
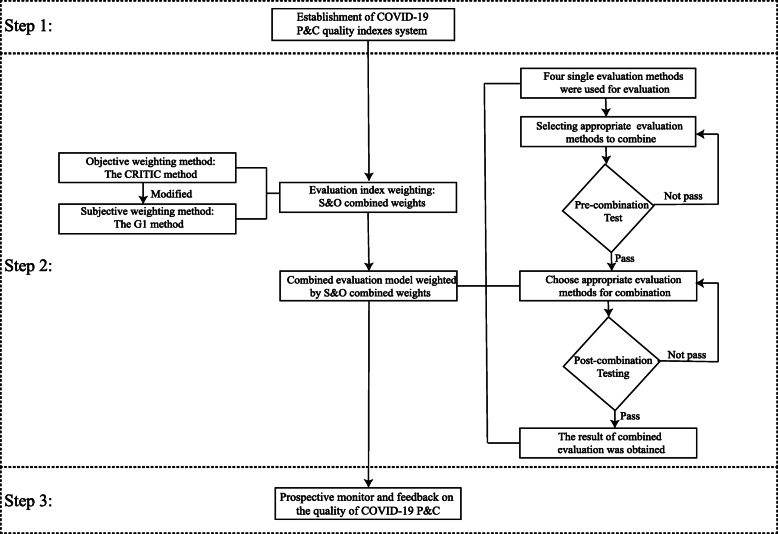
The study flow chart. Note. G1 Order Relation Analysis, CRITIC Criteria Importance Though Intercriteria Correlation, COVID-19 Corona Virus Disease 2019, S&O Subjective and Objective, P&C Prevention and Control

Then, the comprehensive evaluation value was obtained by four methods weighted by the S&O combined weights: weighted EC method, weighted TOPSIS method, weighted RSR method, and weighted GRA method. In addition, the methods of pre and post combination tests evaluated the consistency of the results. Finally, the linear normalization method was used to synthesize the outcomes of various evaluation methods to obtain the value of COVID-19 P&C quality in critical departments. The third step was the prospective monitoring, evaluation, and feedback step. This evaluation method was used to prospectively monitor and evaluate the COVID-19 P&C quality in the critical department from April 2020 to May 2021. The evaluation results were reported to the departments monthly. The study flow chart is shown in Fig. 1.

### Establishment of evaluation indexes system

The modified Delphi method [14] was adopted. First of all, all indicators of COVID-19 P&C quality evaluation were preliminarily listed through national diagnosis and treatment guidelines [15], P&C plans [16], and relevant literature search [17], including three first-level indicators, 17 second-level indicators, and 48 third-level indicators. Then, an expert consulting group consisting of 11 people was established, including three people in the field of infection and control, two people in the area of medical treatment, two people in the field of nursing, two people in the field of epidemiology, and two people in the field of medical laboratory science. All the experts were from our hospital, and had been engaged in relevant profession for more than 10 years, and were proficient in their profession. An expert consultation table was prepared, and the Likert 5-level scoring method [18] was used to score the purpose, completeness, operability, and independence [19] of the indicators one by one. Each attribute of the indicators with 1–5 points was successively increased. Experts ranked the importance of the indicators on a scale of 1 to 10. The experts self-evaluated the judgment basis and familiarity of the indicators by using the pre-established scoring criteria. An expert supplementary opinion column is designed in the consultation table, and experts can add, modify, and delete various indicators. The number of indicators removed by each expert must be less than 50% of the total indicators. Finally, the threshold value method was used to screen the evaluation indexes. The full mark frequency, the arithmetic mean, and the coefficient of variation were calculated according to each index’s scores.

The calculation method of the threshold value for full mark frequency and score’s arithmetic mean are as follow:

1 $$ \mathrm{threshold}\ \mathrm{value}=\mathrm{mean}\ \mathrm{value}-\mathrm{standard}\ \mathrm{deviation} $$

The calculation method of the threshold value for score variation coefficient is as follow:

2 $$ \mathrm{threshold}\ \mathrm{value}=\mathrm{mean}\ \mathrm{value}+\mathrm{standard}\ \mathrm{deviation} $$

Indicators with scores below the threshold value were retained and vice versa. To prevent essential indicators from being removed, only three scales that did not meet the criteria at the same time would be released. If one or two scales did not meet the criteria, the choice should be made after a discussion based on the principles of purpose, completeness, operability, and independence.

After two rounds of expert consultation, the quality evaluation indexes system of COVID-19 P&C was finally determined. The expert consultation results were measured by the expert positive coefficient (Response rate), degree of expert opinion concentration (Mean value), degree of specialist opinion coordination (Coefficient of variation and coordination), and expert authority coefficient (Cr). The Cr is the arithmetic mean of the judgment coefficient (Cs) and the familiarity coefficient (Ca). The calculation formula is as follow:

3 $$ \mathrm{Cr}=\left(\mathrm{Ca}+\mathrm{Cs}\right)/2 $$

### Indicators data collection

The process indicators, such as the HH compliance rate, consumption of HH products, awareness rate of COVID-19 P&C knowledge, and compliance rate of various infection control measures, resulted from manual investigation and monitoring conducted by professionally trained infection control professionals twice a week. Moreover, the outcome indicators, such as the incidence rate of nosocomial infections and the number of occupational exposure cases, were derived from the nosocomial infection information system. The system has a data interface with the hospital information system (HIS), laboratory information system (LIS), radiology information system (RIS), mobile nursing system, operating room anesthesia information system, and could obtain relevant data.

### The objective weighting of indicators (CRITIC method)

The CRITIC method is an objective weighting method proposed by Diakoulaki [20]. Its basic idea is to determine the objective weight of indicators based on two fundamental concepts. The first is the contrast intensity, which represents the value difference of each evaluation object in the same index and is expressed in the form of standard deviation. The larger the standard deviation, the more significant the value difference of each evaluation object. The second is the conflict between indicators, which is based on the correlation between indicators. If there is a strong positive correlation between two indicators, the conflict between two indicators is low.

The quantification index of conflict between the j index and other indexes is:

4 $$ {\sum}_{t=1}^n\left(1-{r}_{tj}\right) $$

where r_tj_ is the correlation coefficient between the evaluation indexes t and j.

Therefore, the conflict and correlation of data are considered more comprehensively in this method than in other objective weighting methods. Let the C_j_ represents the information content contained in the j evaluation index, then C_j_ can be expressed as:

5 $$ {\mathrm{C}}_j={\delta}_j{\sum}_{t=1}^n\left(1-{r}_{tj}\right) $$

Where j = 1,2, …, m; the *δ*_*j*_ represents the standard deviation of the j valuation index.

The larger the C_j_ is, the more information in the j evaluation index is, and the greater the relative importance is. Therefore, the objective weight of the j index is as follows:

6 $$ {\theta}_j=\frac{C_j}{\sum_{j=1}^m{C}_j} $$

Where j = 1,2, …, m.

### Adjusting the calculation of subjective weight with objective weight

The G1 method is a subjective weighting method proposed by Professor Yajun G through improved the AHP method [21]. Compared with AHP, it is no need for a consistency test. And it solves the problem that it is difficult to judge and reach a consensus on the relative importance of indexes in AHP when there are many indexes.

The order relation of each index was determined by each index weight obtained during the establishment of the evaluation indexes system: *x*_1_ > *x*_2_ > … > *x*_*m*_. Then, the objective weights obtained above were used as the comparative judgment of the relative importance between *x*_*k* − 1_ and *x*_*k*_:

7 $$ {r}_k=\frac{w_{k-1}}{w_k} $$

Where *k* = *m*, *m* − 1, ⋯, 3, 2.

8 $$ {r}_k=\left\{\begin{array}{c}\min \left\{2,{w}_{k-1}/{w}_k\right\},\kern2.75em {w}_{k-1}>{w}_k\\ {}1,\kern10.25em {w}_{k-1}<{w}_k\end{array}\right. $$

Then the combined weight of the m index was:

9 $$ {v}_m={\left(1+{\sum}_{k=2}^m{\prod}_{j=k}^m{r}_j\right)}^{-1} $$

From the *v*_*m*_, the m-1,m-2…, 3, 2 indicator weights were:

10 $$ {v}_{j-1}={r}_j{v}_j $$

Where j = m, m-1, m-2, …, 3, 2.

In the formula, the *v*_*j* − 1_ was the G1 combined weight modified by the objective weight of the j-1 index, the *r*_*j*_ was the ratio calculated through Eq. (8), and the *v*_*j*_ was the G1 combined weight modified by the objective weight of the j index.

### Calculation of comprehensive evaluation value

#### Weighted GRA method [22]

The GRA was established and developed by Professor Deng Julong from Huazhong University of Science and Technology in the 1980s. Its basic idea is to distinguish the correlation degree by analyzing and comparing the influence of the sequence index’s change on the reference index. The analysis steps are as follows:

1. Determine the reference sequence

According to the meaning of the evaluation index, the optimal value of each index is selected from n evaluated objects to form a reference sequence *x*_0*j*_. If j is a positive indicator, the bigger, the better, then *x*_0*j*_ is the maximum value of the j index of the n evaluation object; if it is a reverse indicator, it is the minimum value; if it is a moderate indicator, it is the moderate value of the index.

(2) Calculate two-level maximum difference ∆(Max) and two-level minimum difference ∆(min)

Calculate the absolute difference sequence between each evaluation object sequence and the optimal reference sequence, and the calculation formula is as follows:

11 $$ {\Delta }_{ij}=\left|{x}_{ij}-{x}_{0j}\right| $$

Where i = 1,2, …,n, j = 1,2, …,p.

On this basis, according to Eqs. (12) and (13), two-level maximum ∆(Max) and two-level minimum difference ∆(min) can be obtained.

12 $$ \Delta  \left(\max \right)=\mathrm{maxmax}\left({\Delta }_{ij}\right) $$

13 $$ \mathrm{and}\Delta  \left(\min \right)=\mathrm{minmin}\left({\Delta }_{ij}\right) $$

(3) Calculate the correlation degree

The correlation coefficient is calculated according to the formula:

14 $$ {\tau}_{ij}=\frac{\Delta  \left(\min \right)+\uprho \Delta  \left(\mathit{\max}\right)}{\Delta _{ij}+\rho \Delta  \left(\max \right)} $$

Where the ρ (0 < ρ < 1) is the resolution coefficient, and the ρ for this study was 0.05.

(4) Calculate the grey correlation degree according to the formula:

15 $$ {E}_i=\frac{1}{n}{\sum}_{i=1}^n{w}_j{\tau}_{ij} $$

Where *w*_*j*_ is the weight of item j.

(5) Permutation correlation degree

Since the *E*_*i*_ reflects the degree of correlation between the i evaluated object and the evaluation standard sequence *x*_0*j*_, if *E*_*i*_ > *E*_*j*_, it indicates that the i object is better than the j object. So the *E*_*i*_ can sort and compare the evaluated objects.

#### Weighted EC method [23]

EC method also called the efficiency function method, is based on the principle of multi-objective programming to determine a satisfactory value and a disallowed value for each evaluation index, with the satisfactory value as the upper limit and the disallowed value as the lower limit. The degree of each indicator’s satisfaction is calculated. Then the score of each indicator is determined based on this. The comprehensive status of the studied object is evaluated through the weighted average. The calculation steps are as follows:

1. Determine the satisfactory value and the disallowable value of each evaluation index.

The satisfaction value of the index is χ_hij_ (i = 1,2…, n. j = 1,2…, m) and the unallowable values of the index is χ_sij_ (i = 1,2…, n. j = 1,2…, m).

(2) Calculate the EC of each index according to this formula:

16 $$ {d}_{ij}=\frac{x_{ij}-{x}_{sij}}{x_{hij}-{x}_{sij}}\times 40+60 $$

Where the d_ij_ represents the i (i = 1, 2, …, n) EC values of the j (j = 1,2…m) evaluation indexes. The *x*_*ij*_ represents the i (i = 1, 2, …, n) measurement values of the j (j = 1, 2, …, m) evaluation indexes.

(3) The total EC of each evaluation object is calculated:

17 $$ {D}_i={\prod}_i^m{d}_{ij}^{w_j} $$

Where *D*_*i*_ represents the total EC value of the i (i = 1,2…, n) evaluation object; The d_ij_ denotes the j (j = 1,2…, m) power coefficient values of the i (i = 1,2…, n) evaluation indexes; The w_j_ represents the j (j = 1,2…m) weight of evaluation indexes.

The evaluation object is evaluated according to the total EC value. Thus, the greater the total EC value is, the better the comprehensive condition of the evaluation object is.

#### Weighted RSR method [24]

The method of RSR was proposed by Professor Tian Fengtian, a Chinese statistician, in 1988. The statistical analysis method, which combines the advantages of classical parametric statistics and modern non-parametric statistics, is not only suitable for the comprehensive evaluation of four-grid data but also for row × table data, as well as for metrological data and classified data. The calculation steps are as follows:

1. Rank compiling

The rank of the high superior index is ranked from small to large. That is, the minimum value of the index is ranked as rank 1, the small value of the index is ranked as rank 2, …, the maximum value of the index is ranked as rank n; On the contrary, the low superior index varies from large to small rank. The mean rank is used for those with the same index value. The average rank is used for the intermediate superior index.

(2) The weighted RSR is calculated:

18 $$ {WRSR}_i=\frac{1}{n}{\sum}_{j=1}^m{W}_j{R}_{ij} $$

Where the i = 1,2…, n, j = 1,2…, m, the R_ij_ represents the rank of the element in row i and column j, and the W_j_ means the weight of the j evaluation index.

The value of WRSR is used to determine the merits and demerits of the evaluation object. Thus, the larger the WRSR is, the better the evaluation object is.

#### Weighted TOPSIS method [25]

C.L. Hwang and K. Yue first proposed the TOPSIS method in 1981. TOPSIS is a method to rank a limited number of evaluation objects according to their proximity to the ideal goal, which is to evaluate the relative merits of existing objects. The calculation steps are as follows:

1. Indexing the same trend

The counting backward technique is used to change the low superior indexes to high superior indexes.

(2) The dimensionless of the indexes

After the same trend, the original data matrix is normalized according to the following formula to eliminate the influence of indexes’ measurement and establish a normalized matrix *Ζ*.

When the original data is a high superior indicator:

19 $$ {Z}_{ij}=\frac{x_{ij}}{\sqrt{\sum_{i=1}^n{x}_{ij}^2}} $$

When the original data is a low superior indicator:

20 $$ {Z}_{ij}=\frac{x_{ij}^{\prime }}{\sqrt{\sum_{i=1}^n{\left({x}_{ij}^{\prime}\right)}^2}} $$

(3) Determine positive and negative ideal solutions

According to the normalized matrix *Ζ*, the positive ideal solution (optimal vector) is obtained:

21 $$ {Z}^{+}=\left({Z}_{i1}^{+},{Z}_{i2}^{+},\cdots, {Z}_{im}^{+}\right) $$

And the negative ideal solution:

22 $$ {Z}^{-}=\left({Z}_{i1}^{-},{Z}_{i2}^{-},\cdots, {Z}_{im}^{-}\right) $$

Where i = 1,2…, n. j = 1,2…, m. ${Z}_{i1}^{+}$ + and ${Z}_{i1}^{-}$ represent the maximum and minimum value of the evaluation object in the j index, respectively.

(4) Calculate the Euclide distance

${D}_i^{+}$ + and ${D}_i^{-}$ between the index values of each evaluation object and the positive and negative ideal solutions:

23 $$ {D}_i^{+}=\sqrt{\sum_{j=1}^m{\left[{w}_j\left({Z}_{ij}-{Z}_{ij}^{+}\right)\right]}^2} $$

24 $$ {D}_i^{-}=\sqrt{\sum_{j=1}^m{\left[{w}_j\left({Z}_{ij}-{Z}_{ij}^{-}\right)\right]}^2} $$

Where the *w*_*j*_ represents the weight of index j.

(5) Calculate the C_i_ value

The C_i_ value of the relative proximity between the index value of each evaluation object and the positive and negative ideal solution is calculated:

25 $$ {C}_i=\frac{D_i^{-}}{D_i^{+}+{D}_i^{-}} $$

According to the C_i_’s relative proximity coefficient, the order of the merits and demerits of evaluation objects is sorted. The value range of C_i_ is [0,1]. The closer the C_i_ value is to 1, the closer the evaluation object is to the positive ideal solution. Conversely, the closer the C_i_ value is to 0, the further away the evaluation object is from the positive ideal solution.

### Pre-combination test

The Kendall rank correlation [26] was used to test whether the ranking results of every single evaluation method are consistent. Only evaluation methods that passed the pre-combination test could be used for combinations.

### Synthesis of comprehensive evaluation results (information synthesis method)

#### Constructing the matrix of comprehensive evaluation results

The results obtained from the comprehensive evaluation method passed by the pre-combination test, such as total EC, WRSR, C_i_ value, and GR degree, are summarized to form the evaluation result matrix. The evaluation result matrix is marked by (*x*_*ij*_)_*m* × *n*_, where *x*_*ij*_ represents the evaluation result of the j object by the i evaluation method. The m represents m evaluation methods, and the n represents n evaluation objects.

#### Equation (26) is used to linearize the evaluation result matrix by row

26 $$ {y}_{ij}=\frac{x_{ij}-{\min}_{j=n}^n{x}_{ij}}{\max_{j=1}^n{x}_{ij}-{\min}_{j=1}^n{x}_{ij}} $$

Where *y*_*ij*_ represents the value of each evaluation object after normalization.

#### Calculate the mean normalized values of each evaluation object according to Eq. (27)

27 $$ {\overline{y}}_j={\sum}_{i=1}^m\frac{y_{ij}}{m} $$

Rearrange according to the size of ${\overline{y}}_j$ value. The larger the value is, the better the evaluation object is.

### Post-combination test

The Spearman rank correlation test method [26] was used to test the degree of closeness between the ranking results obtained after the combination and the results obtained from the initial method. If the order of the combined evaluation method was inconsistent with one of the evaluation methods in the combination, the method should be eliminated and then recombined.

### Statistics and analysis

Input the data into Excel 365 to establish the database. In excel, The indexes weighting and comprehensive evaluation were completed, and the Mann-Kendall [27] was used to test the trend of critical departments’ comprehensive evaluation value. Descriptive analysis, Kendall consistency test, and Spearman rank correlation test were performed using SPSS 26.0 (Armonk, NY, USA: IBM Corp) software. Continuity variables conforming to normal distribution were described by mean ± standard deviation. The non-normal distribution data was described by the median (interquartile interval). *P*-values of less than 0.05 were considered significant.

## Results

### Establishment of COVID-19 P&C quality evaluation indexes system

After two rounds of letter consultation by 11 experts (average age: 36 ± 3.5 years), the indexes system for the quality evaluation of COVID-19 P&C was finally determined, including one first-level indicator, eight second-level indicators, and 13third-level indicators. The composition and the calculation formula of the indicators are shown in Table 1.

**Table 1:** Composition of COVID-19 control quality index system

First-level indicators	Second-level indicators	Third-level indicators	Formula
Quality of COVID-19 P&C	X_1_, Material support	X_11_, Sufficient rate of PPE (%)	NO. of PPE prepared÷NO. of PPE required× 100%
		X_12_, Adequacy rate of equipment and facilities (%)	Inspection pass NO.÷Inspection NO. × 100%
	X_2_, Patient management	X_21_, Standardized patient management rate (%)	Inspection pass NO.÷Inspection NO. × 100%
	X_3_, Sterile supplies management	X_31_, Standard rate of sterile supplies management (%)	Inspection pass NO.÷Inspection NO. × 100%
	X_4_, Sterilization	X_41_, Qualified rate of environmental cleaning and disinfection (%)	Monitoring pass NO.÷Monitoring NO. × 100%
	X_5_, HH	X_51_, HH compliance rate (%)	NO. of HH÷NO. of HH opportunities×100%
		X_52_, Correct rate of HH (%)	NO. of correct HH÷NO. of HH × 100%
	X_6_, occupational protection	X_61_, Correct use rate of PPEs (%)	Inspection pass NO.÷Inspection NO. × 100%
		X_62_, Occupational protection knowledge awareness rate (%)	NO. of qualified÷NO. of trainees×100%
	X_7_, training and education	X_71_, Training rate of COVID-19 P&C knowledge of staff (%)	NO. of trainees÷NO. of HcWs×100%
		X_72_, Qualified rate of COVID-19 P&C knowledge assessment (%)	NO. of qualified HcWs÷NO. of HcWs assessed×100%
	X_8_, Sewage disposal	X_81_, Standard rate of medical waste disposal (%)	Inspection pass NO.÷Inspection NO. × 100%
		X_82_, Disposal standard rate of contaminated fabric (%)	Inspection pass NO.÷Inspection NO. × 100%

*COVID-19* Corona Virus Disease 2019, *NO.* Number, *PPE* Personal Protective Equipment, *HH* Hand Hygiene, *HcWs* Healthcare Workers, *P&C* Prevention and Control

In the first round of expert consultation, 11 questionnaires were sent out, ten were returned, and all of them were valid, with a response rate of 90.91% and a valid rate of 100%. Six experts put forward a total of 12 opinions. Ten questionnaires were sent out in the second round, ten were returned, and all were valid. The response rate and valid rate were all 100%. A total of four opinions were put forward by three experts, which indicated that experts were more active in the two rounds of letter consultation. The authority coefficients of the two rounds of expert consultation were all greater than 0.8, indicating that the authority of the experts selected in this study was relatively high, and the results had certain representativeness. The Kendall consistency test showed that the coordination coefficient greater than 0.3 (*P* < 0.0001) indicated a strong evaluation consistency. The mean value, standard deviation, and full mark ratio are shown in Table 2. Indicator selection was determined by the threshold values of full mark frequency, mean value, and coefficient of variation in the two rounds of letters consultation. The values of the two rounds are shown in Table 3.

**Table 2:** Delphi expert authority coefficient, degree of opinion concentration and degree of opinion coordination

Survey rounds	Cs	Ca	Cr	Mean (min-max)	Sd. (min-max)	Full score ratio (min-max)	Kendall coordination coefficient	χ^2^	*P*
First-round	0.679	0.930	0.805	2.575–4.325	0.230–1.125	7.50–55.00%	0.399	191.353	<0.001
Second-round	0.715	0.980	0.848	2.725–4.125	0.230–1.080	10.00–40.00%	0.378	151.220	<0.001

*Cr* Expert Authority Coefficient, *Cs* Judgment Coefficient, *Ca* Familiarity Coefficient, *Sd.* Standard Deviation

**Table 3:** The threshold values used for indexes screening in the two rounds of the Delphi method

Screening program	First-round			Second-round		
	Mean	Sd.	Threshold value	Mean	Sd.	Threshold value
Full mark frequency (%)	24.643	9.214	15.429	24.390	7.305	17.085
Mean	3.280	0.479	2.801	3.336	0.473	2.863
CV	0.197	0.076	0.273	0.197	0.088	0.285

*Sd.* Standard Deviation, *CV* Coefficient of Variation

### The combination of the S&O weighting of evaluation indexes

According to the data in March 2020, the indicators’ objective weights were obtained, and the subjective weight calculation process of the G1 method was adjusted with the objective weight. As a result, the combined S&O weights of each evaluation indicator were obtained. The weighted rankings of each index of the combined weight were the same as the subjective weighting method, reflecting the personal wills. In addition, the size of the combined weight was fine-tuned by the objective weight, reflecting the nature of the data. The weighting process of the S&O combination is shown in Table 4.

**Table 4:** S&O combined weights of each evaluation index

Evaluation index	The importance rankings of indicators by the G1 method	Objective Weights (CRITIC method)	The ratio of the importance of indicators	Combination of S&O weights
X11, Sufficient rate of PPE	2	0.0655	1.0000	0.1079
X_12_, Adequacy rate of equipment and facilities	4	0.0752	1.0845	0.0995
X_21_, Standardized patient management rate	1	0.0642	\	0.1079
X_31_, Standard rate of sterile supplies management	10	0.0622	1.2391	0.0544
X_41_, Qualified rate of environmental cleaning and disinfection	9	0.0771	1.0000	0.0674
X_51_, HH compliance rate	8	0.0766	1.0143	0.0674
X_52_, Correct rate of HH	11	0.0697	1.0000	0.0544
X_61_, Correct use rate of PPE	3	0.0815	1.0000	0.1079
X_62_, Occupational protection knowledge awareness rate	7	0.0777	1.2173	0.0684
X_71_, Training rate of COVID-19 P&C knowledge of HcWs	6	0.0946	1.0000	0.0832
X_72_, Qualified rate of COVID-19 P&C knowledge assessment	5	0.0629	1.1955	0.0832
X_81_, Standard rate of medical waste disposal	13	0.0860	1.2419	0.0438
X_82_, Disposal standard rate of contaminated fabric	12	0.1069	1.0000	0.0544

*G1* Order Relation Analysis, *CRITIC* Criteria Importance Though Intercriteria Correlation, *PPE* Personal Protective Equipment, *HH* Hand Hygiene, *COVID-19* Corona Virus Disease 2019, *S&O* Subjective and Objective, *P&C* Prevention and Control

### The evaluation results of each comprehensive evaluation method

The evaluation results of the weighted EC method, weighted RSR method, weighted TOPSIS method, and weighted GRA method for each evaluation object were different, as shown in Table 5.

**Table 5:** Evaluation results of each comprehensive evaluation method

Departments	Weighted EC method		Weighted RSR method		Weighted TOPSIS method		Weighted GRA degree	
	Total EC	Rankings	RSR	Rankings	C value	Rankings	GR degree	Rankings
CT Room	58.072	8	0.592	6	0.492	6	0.632	9
ICU	58.612	4	0.550	8	0.460	9	0.678	5
Delivery room	54.641	13	0.440	12	0.382	11	0.617	11
Pediatrics	56.997	11	0.426	13	0.367	14	0.606	12
Department of ENT	58.240	6	0.595	5	0.466	8	0.687	4
Infectious diseases department	58.016	9	0.556	7	0.495	5	0.713	1
Infectious disease clinic	58.295	5	0.515	9	0.518	2	0.636	8
ED	59.307	3	0.627	3	0.502	4	0.661	7
Stomatology department	55.073	12	0.480	10	0.379	12	0.606	13
Endoscope room	59.865	2	0.634	1	0.620	1	0.711	2
General clinic	58.135	7	0.604	4	0.483	7	0.699	3
OR	57.369	10	0.477	11	0.401	10	0.623	10
Hemodialysis room	53.032	14	0.376	14	0.377	13	0.574	14
Ophthalmology department	60.186	1	0.627	2	0.517	3	0.675	6

*EC* Efficacy Coefficient, *TOPSIS* Technique for Order Preference by Similarity to an Ideal Solution, *RSR* Rank-Sum Ratio, *GRA* Grey Relationship Analysis, *CT* Computerized Tomography, *ICU* Intensive Care Unit, *ENT* Ear-Nose-Throat, *ED* Emergency Department, *OR* Operating Room

### Pre-combination test

The Kendall consistency test of the four comprehensive evaluation methods on the evaluation objects showed the Kendall synergy coefficient was 0.835 (χ^2^ = 43.429, *P* < 0.001), more significant than 0.8. Thus, the evaluation consistency of the four methods was good, and the results of the four evaluation methods could be integrated.

### Synthesize the results of various comprehensive evaluation methods

After linear normalization, the mean value of evaluation results was obtained. Then the P&C quality of COVID-19 in each department was ranked by the mean value. The top three departments were endoscopy, ophthalmology, and emergency department, while the bottom three departments were delivery room, stomatology, and pediatrics, as shown in Table 6.

**Table 6:** Rankings of evaluation objects synthesized by information synthesis method

Departments	The linear normalization of each evaluation method results				Mean value	Rankings
	Weighted EC method	Weighted RSR method	Weighted TOPSIS method	Weighted GRA method		
CT room	0.704	0.835	0.496	0.420	0.614	8
ICU	0.780	0.673	0.367	0.744	0.641	7
Delivery room	0.225	0.249	0.061	0.308	0.211	13
Pediatrics	0.554	0.193	0.000	0.234	0.245	11
Department of ENT	0.728	0.847	0.393	0.808	0.694	6
Infectious diseases department	0.697	0.696	0.505	1.000	0.725	5
Infectious disease clinic	0.736	0.539	0.598	0.449	0.580	9
ED	0.877	0.970	0.536	0.623	0.751	3
Stomatology department	0.285	0.403	0.049	0.230	0.242	12
Endoscope room	0.955	1.000	1.000	0.985	0.985	1
General clinic	0.713	0.884	0.460	0.898	0.739	4
OR	0.606	0.392	0.134	0.355	0.372	10
Hemodialysis room	0.000	0.000	0.040	0.000	0.010	14
Ophthalmology department	1.000	0.973	0.595	0.725	0.823	2

*EC* Efficacy Coefficient, *TOPSIS* Technique for Order Preference by Similarity to an Ideal Solution, *RSR* Rank-Sum Ratio, *GRA* Grey Relationship Analysis, *CT* Computerized Tomography, *ICU* Intensive Care Unit, *ENT* Ear-Nose-Throat, *ED* Emergency Department, *OR* Operating Room

### Post-combination test

The correlation coefficients between the combined evaluation method and each evaluation method were above 0.8, with statistical significance, as shown in Table 7.

**Table 7:** Correlation coefficient and the *P*-value of combined evaluation method and each evaluation method

Evaluation methodology	Weighted EC method	Weighted RSR method	Weighted TOPSIS method	Weighted GRA method
Combined evaluation method	0.886	0.956	0.811	0.855
*P*	< 0.001	< 0.001	< 0.001	< 0.001

*EC* Efficacy Coefficient, *TOPSIS* Technique for Order Preference by Similarity to an Ideal Solution, *RSR* Rank-Sum Ratio, *GRA* Grey Relationship Analysis

### The quality trend of COVID-19 P&C in critical departments

**Fig. 2:**
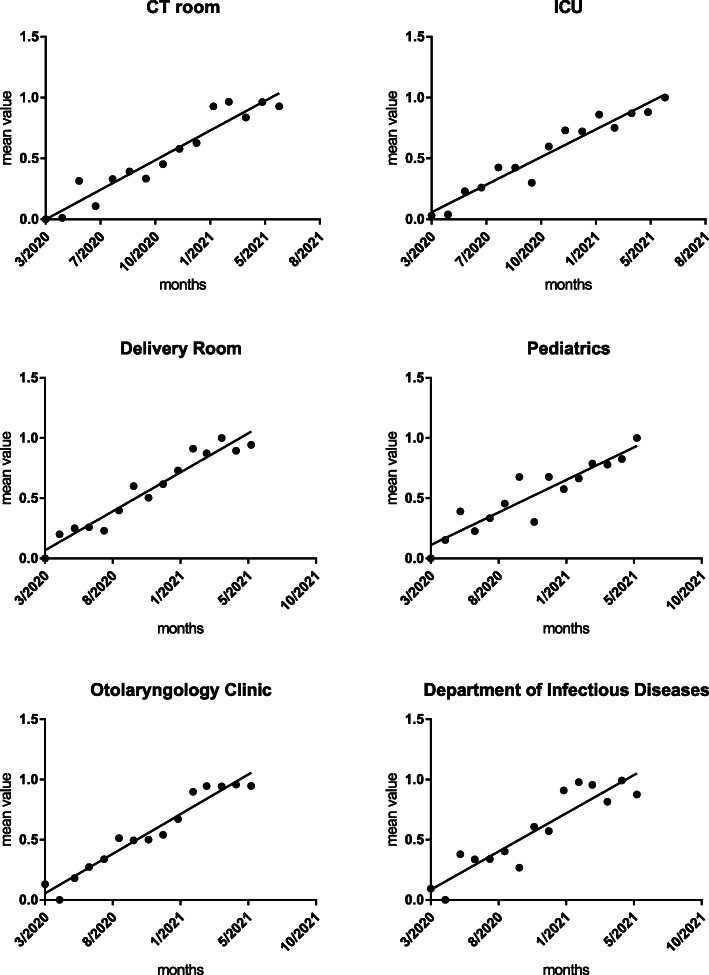
Trend chart of the mean quality value of COVID-19 P&C in crucial departments. The black dots represent the comprehensive evaluation mean value of COVID-19 P&C for the critical department. The solid line represents the trend prediction for the comprehensive evaluation mean value. Note. CT Computerized Tomography, ICU Intensive Care Unit

**Fig. 3:**
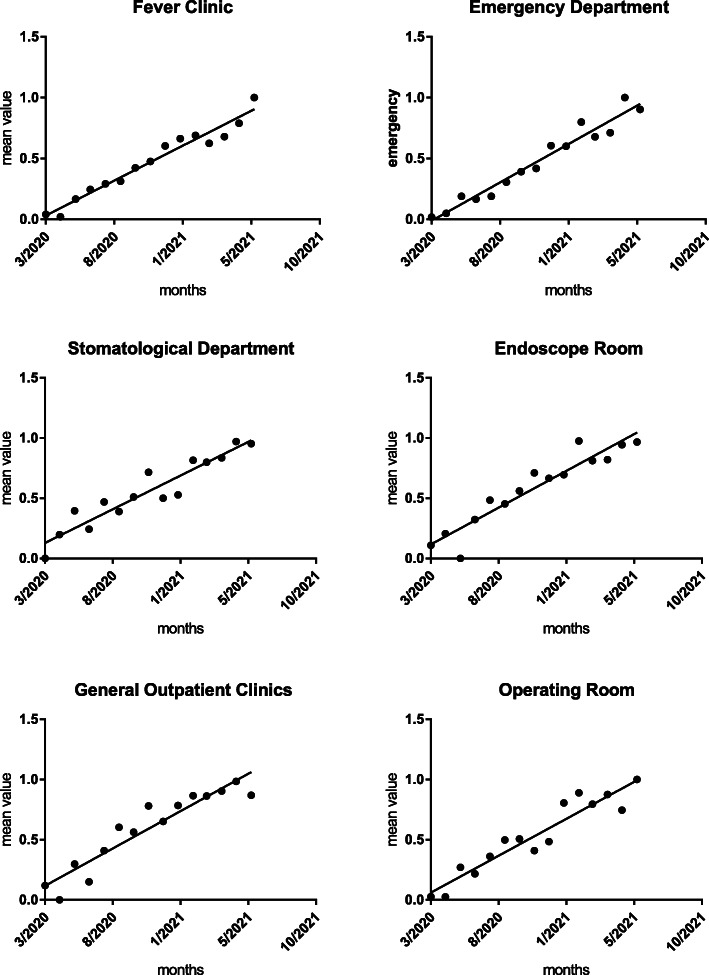
Trend chart of the mean quality value of COVID-19 P&C in crucial departments. The black dots represent the comprehensive evaluation mean value of COVID-19 P&C for the critical department. The solid line represents the trend prediction for the comprehensive evaluation mean value

**Fig. 4:**
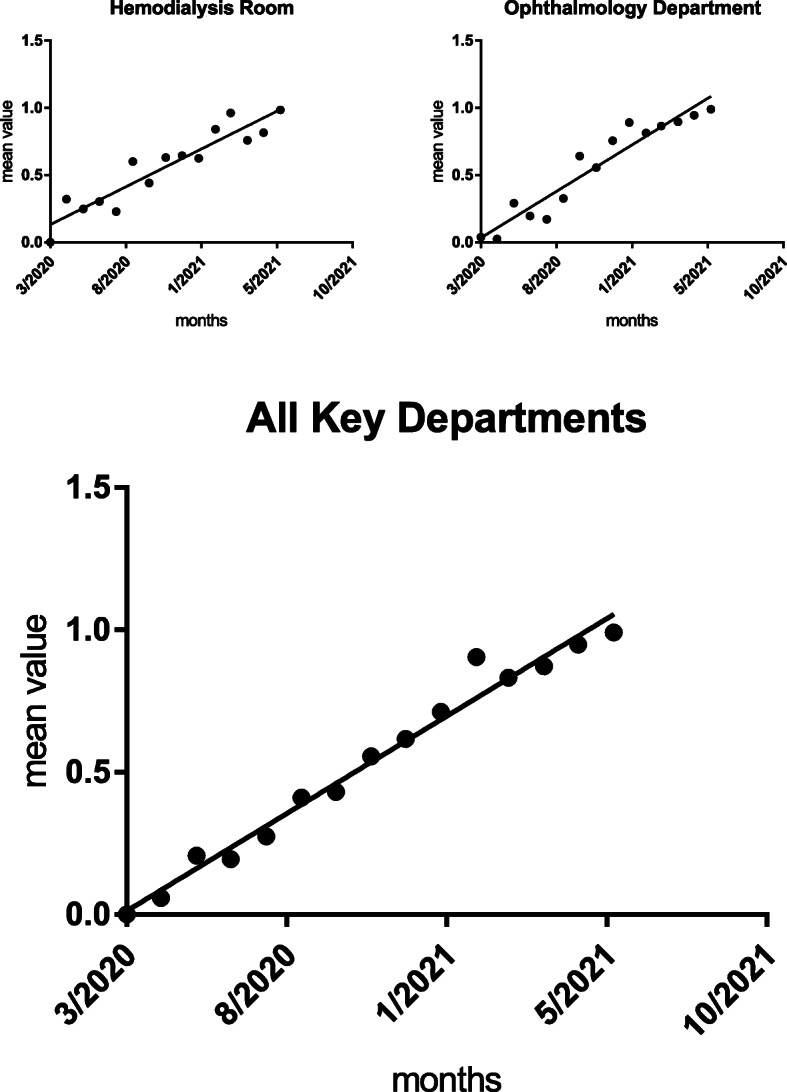
Trend chart of the mean quality value of COVID-19 P&C in crucial departments. The black dots represent the comprehensive evaluation mean value of COVID-19 P&C for the critical department. The solid line represents the trend prediction for the comprehensive evaluation mean value

The mean quality value showed an upward trend by the Mann Kendall test, as shown in Table 8, Figs. 2, 3, and 4.

**Table 8:** Mann Kendall test results of the mean quality value in critical departments

Departments	Slope (*β)*	*Z*-value	*P*-value
CT room	0.074	4.355	< 0.01
ICU	0.069	4.652	< 0.01
Delivery room	0.070	4.454	< 0.01
Pediatrics	0.059	4.058	< 0.01
Department of ENT	0.071	4.652	< 0.01
Infectious diseases department	0.069	3.563	< 0.01
Infectious disease clinic	0.062	4.751	< 0.01
ED	0.069	4.652	< 0.01
Stomatology department	0.061	4.355	< 0.01
Endoscope room	0.066	4.256	< 0.01
General clinic	0.067	4.454	< 0.01
OR	0.067	3.860	< 0.01
Hemodialysis room	0.061	3.959	< 0.01
Ophthalmology department	0.075	4.454	< 0.01
All critical department	0.074	4.850	< 0.01

*CT* Computerized Tomography, *ICU* Intensive Care Unit, *ENT* Ear-Nose-Throat, *ED* Emergency Department, *OR* Operating Room

## Discussion

COVID-19 P&C is currently one of the critical tasks in most countries and regions. The essential point of P&C work is implementing the scientific P&C norms and guidelines. However, to evaluate the quality of implementation, a single evaluation index is often not comprehensive enough. Multiple indicators are difficult to judge the overall situation. Therefore, multiple indicators should be integrated to obtain a scientific and comprehensive evaluation result [28].

An essential function of the multi-index comprehensive evaluation method is “dimensionality reduction,” that is, many index values are integrated into a single evaluation value to facilitate comparison. However, if too many indicators are involved in the evaluation, a correlation may exist, affecting the results. It also increases the workload and difficulty in the assessment. Therefore, it was necessary to choose the representative index by discarding the dross and selecting the essential to establish a scientific evaluation system. This study thoroughly considered the evaluation indexes’ purpose, completeness, operability, and independence through two rounds of the Delphi letter consultation method. Therefore, the opinions of evaluation experts could be consistent and unified to establish a quality evaluation indexes system of COVID-19 P&C in line with the actual situation of our hospital.

At present, there are more than ten commonly used methods of the multi-index comprehensive evaluation methods, such as expert opinion method, Delphi method, AHP method, G1 method, coefficient of variation method, EC method, entropy method, maximum deviation method, RSR method, TOPSIS method, GRA method, principal component analysis method, factor analysis method, and fuzzy evaluation method. Different evaluation methods may obtain different results due to diverse principles and the sensitivity of indexes to evaluation methods. Therefore, it is not possible to use only one evaluation method for evaluation. For minimizing the impact of evaluation methods on evaluation results, four methods, including the weighted EC method, weighted RSR method, weighted TOPSIS method, and weighted GRA method, were adopted in this study to evaluate the quality of COVID-19 P&C in critical departments. The evaluation results of different methods were reasonably integrated, and the pre and post combination testing ensured the reliability and stability of the results.

Traditional weight combination methods generally include 1. Addition synthesis methods, such as the linear weighting method with preference coefficient proposed by Liu X et al. [29], and R. Md Saad et al. [30] proposed the minimum difference method between S&O evaluation information and the ideal scheme. However, the additive synthesis method is too rigid, which is not conducive to treating the superior and inferior information in the S&O weight information and is not conducive to the rational explanation of the combined weights. 2. Multiplication method. For example, Chen Ch [12] used the multiplication method to synthesize the entropy weight and AHP methods to obtain the S&O combined weight. However, the normalization of S&O weights after multiplication is easy to produce a multiplier effect. This results in the combined weights of the indexes with high weights are large. In contrast, the combined weights of the indexes with low weights are small. Which only applies to the situation where the number of indicators is large, and the weight distribution is uniform. Moreover, the significance of combined weights obtained by addition and multiplication methods is not easy to explain. In addition, the combined weighting method of addition and multiplication also needs to solve the allocation of S&O weight coefficients or preference coefficients. There is no ideal solution yet.

According to the research in recent years, many experts have improved the defects of traditional combination weighting. For example, Wenyu Z. et al. [31] based on AHP to stratify the indicators, processed the weights of the indicators by using the two-base point entropy weight method, worked out the objective weight, and obtained the combined weight through the Delphi method. Xin-Xin G. et al. [32] effectively combined the entropy method with AHP to avoid single S&O weighting defects. Yunfei L. et al. [33] proposed a method to calculate the combined weight of indicators based on the principle of vector similarity. First, the importance orders of indicators were determined by the superior belonging degree method of adjacent targets. Then the objective weight of indicators was obtained by the maximum entropy criterion. At last, the final index weight was obtained using vector similarity to find the one closest to the order of subjective weight importance. Gang L. [34] proposed the combined weighting method based on standard deviation and the G1 method, a weighting method that adjusts subjectivity with objectivity. Firstly, the indexes’ importance ranking was given by experts according to their experience, and the superiority of the subjective weighting method was reflected through the ranking. Then, the objective weighting method was used to calculate the indexes’ objective weight. The indexes’ objective weight was used as the basis for calculating the importance ratio between the indexes. And the advantages of the objective weighting method in data information were reflected by calculating the importance ratio of adjacent indexes. Finally, the G1 weighting method was used to calculate the combined S&O weights.

These weighting methods solved the mechanical combination of S&O weights in the traditional weighting methods. Therefore, there is no need to determine the preference coefficient of S&O weights when combining. Thus, at present, they are relatively reasonable combination methods. However, the standard deviation only considered the conflict between the data, didn’t consider the correlation of the data. Therefore, to entirely use the data’s nature, this study used the CRITIC objective weighting method to integrate the conflict and correlation of data.

As an effective measure of quality improvement, monitoring and feedback were widely used in nosocomial infection P&C practice. For example, the monitoring and feedback on the HH of HcWs were helpful to improve the HH compliance rate [35]. In addition, monitoring and feedback of the implementation rate of P&C measures for catheter-associated urinary tract infections were conducive to reducing urinary tract infections in patients [36]. In this study, monitoring and feedback were used to monitor the quality of COVID-19 P&C work in critical departments monthly. The rankings of work quality were timely fed back to departments. The longitudinal evaluation of the key departments showed that the mean value of the combined assessment showed an upward trend, suggesting that monitoring and feedback of the quality of COVID-19 P&C could promote the improvement of the P&C quality.

This study used a scientific and rigorous method to establish a quality evaluation index system for COVID-19 P&C. The combination weight was obtained through the subjective weight modified by the objective weight, which avoided the defects of the addition and multiplication combination method. The index’s sensitivity to the method was reduced by using various data synthesis methods to obtain scientific and stable evaluation results. However, there were still some shortcomings in this study. First of all, the quality indexes system of COVID-19 P&C, in line with the actual situation of our hospital, was established through two rounds of the Delphi method. However, the indicators data were mainly obtained through investigators’ observation, with high subjectivity and low objectivity. Although the investigators were all professional personnel with more than 5 years of experience in infection control and had received unified training before the investigation, observational bias was unavoidable. In addition, no adverse events such as HcWs’ COVID-19 nosocomial infection, occupational exposure to COVID-19, and psychological stress occurrence during the study. Therefore, the indexes system consisted of structural and process indexes, lacking outcome indexes. Then in the combined comprehensive evaluation process, various weighting methods and results-synthesis methods were used. The calculation process was more complex, not convenient for practical application. For this reason, a particular evaluation procedure was developed by Excel’s functions and formulas. When the relevant data were inputted, the results would be obtained automatically to facilitate the practical application of the evaluation method. Finally, this study was an observational study without a control group. The COVID-19 P&C quality in critical departments was monitored and fed back every month in the prospective monitoring step. Although other quality promotion measures had not been implemented, there might still be other confounding factors. A randomized controlled trial needs to be further verified whether monitoring and feedback can improve COVID-19 P&C quality in departments.

## Conclusions

Summarily, in this study, a COVID-19 P&C indexes system with purpose, completeness, operability, independence, and conformity to the actual situation of our hospital was established by the Delphi method. The S&O combined weight was obtained through the G1 weighting adjusted by the CRITIC method, which took into account both the data’s nature and the personal needs. COVID-19 P&C quality in key departments was comprehensively evaluated by the weighted RSR method, weighted EC method, weighted TOPSIS method, and weighted GRA method. The information synthesis method was used to synthesize the comprehensive evaluation results of the four methods reasonably. The combination rationality of the four methods was evaluated by the pre and post combination tests. More scientific and comprehensive evaluation could be achieved in the combined method than the single weighting and single evaluation methods. Finally, this method was used to prospectively monitor and fed back the quality of COVID-19 P&C in critical departments. As a result, COVID-19 P&C quality has been continuously improved through continuous monitoring and feedback, which medical institutions could use as a reference to do excellent COVID-19 P&C works.

## Data Availability

The data used in the study was available from the Tertiary-grade & A-class office of the Shenzhen Hospital of the University of Chinese Academic of Science.

## Abbreviations

COVID-19: Corona Virus Disease 2019
P&C: Prevention and Control
CRITIC: Criteria Importance Though Intercriteria Correlation
G1: Order Relation Analysis
S&O: Subjective and Objective
EC: Efficacy Coefficient
TOPSIS: Technique for Order Preference by Similarity to an Ideal Solution
RSR: Rank-sum ratio
GRA: Grey Relationship Analysis
HH: Hand hygiene
PPE: Personal Protective Equipment
AHP: Analytic Hierarchy Process
MAUT: Multi-Attribute Utility Theory
Cr: Expert authority coefficient
Cs: Judgment coefficient
Ca: Familiarity coefficient
HIS: Hospital Information System
LIS: Laboratory Information System
RIS: Radiology Information System
NO.: Number
HcWs: Healthcare Workers
Sd.: Standard Deviation
CV: Coefficient of Variation
CT: Computerized Tomography
ICU: Intensive Care Unit
ENT: Ear-Nose-Throat
ED: Emergency Department
OR: Operating Room
